# Fluorescence Analysis of Biocide Efficiency in Antifouling Coatings against Cyanobacteria

**DOI:** 10.3390/ijms24054972

**Published:** 2023-03-04

**Authors:** Aleksandra Orzechowska, Anna Czaderna-Lekka, Martin Trtílek, Piotr Rusiniak

**Affiliations:** 1 Faculty of Physics and Applied Computer Science, AGH-University of Science and Technology, Al. Mickiewicza 30, 30-059 Kraków, Poland; 2 Department of Machine Learning, Faculty of Informatics and Communication, University of Economics, 1 Maja 50, 40-287 Katowice, Poland; 3 Photon Systems Instruments, Prumyslova 470, 664 24 Drásov, Czech Republic; 4 Faculty of Geology, Geophysics and Environmental Protection, AGH-University of Science and Technology, Al. Mickiewicza 30, 30-059 Kraków, Poland

**Keywords:** chlorophyll fluorescence, photosystem II (PSII) efficiency, cyanobacteria, *Cyanothece*, toxicity, antifouling coatings

## Abstract

This study focused on the antifouling effect of copper oxide (Cu_2_O)- and zineb-based coatings against *Cyanothece* sp. *ATCC 51142* by analysing photosynthetic activity using chlorophyll fluorescence. The photoautotrophically grown cyanobacterium was exposed to toxic coatings over a short-term period of 32 h. The study showed that *Cyanothece* cultures are particularly sensitive to biocides (i) released from antifouling paints and (ii) exhibited by contact with the coated surfaces. Changes in the maximum quantum yield of photosystem II (F_V_/F_M_) were observed within the first 12 h of exposure to the coatings. Partial recovery of F_V_/F_M_ in *Cyanothece* was revealed 24 h post exposure to a copper- and zineb-free coating. In this research, we proposed an analysis of the evaluation of fluorescence data to study the initial response of cyanobacterial cells to copper- and non-copper-based antifouling coatings formulated with zineb. We evaluated the dynamics of coating toxicity by determining the characteristic time constants of changes in the F_V_/F_M_. Within the most toxic paints studied, those formulated with the highest concentration of Cu_2_O and zineb, the estimated time constants were 3.9 times lower compared to the copper- and zineb-free paint. The use of zineb in copper-based antifouling coatings enhanced the toxic effect of paints and contributed to a faster decline in photosystem II activity in *Cyanothece* cells. The analysis we proposed, along with the fluorescence screening results, may be useful in evaluating the initial antifouling dynamic action against photosynthetic aquacultures.

## 1. Introduction

Biofouling is an undesirable accumulation of organisms on surfaces that are immersed in water and is of great concern for many industries [1]. It causes various industrial problems with significant economic losses. The effects of fouling on boat hulls, ships, or other immersed structures are thoroughly examined [2,3,4,5,6,7]. Fouling affects the hydrodynamic performance of ship hulls as a result of increased weight and frictional resistance. This, in turn, leads to increased fuel consumption [8], higher transportation costs, and also contributes to global climate change [9]. Biofouling is also considered one of the main vectors for the transfer of invasive aquatic species [10]. Although the phenomenon is a well-known and studied problem, the control of bioadhesion remains an economically and ecologically challenging issue [11,12]. Among the most common techniques of eradication with biofouling is the use of toxic compounds in paint matrices, which are then applied to boat hulls to prevent or reduce the growth and colonisation of micro- and macro-organisms. These coatings present an important inhibition effect against the adhesion and growth of bacteria, fungi, microalgae, and cyanobacteria. Antifouling (AF) paints consist of polymeric films, made of mostly acrylic and styrenic monomers, and contain copper or zinc as the active elements. Copper has become the main biocidal component of most AF paints [13]. It usually comes in the form of copper oxide. Copper, being a micronutrient, is essential for life; [14] however, its higher concentration tends to be toxic [15]. The toxicity of copper in water is greatly affected by the chemical form or speciation of the copper and the degree to which it is bound to various ligands present in water [16,17]. When copper from metallic Cu or cuprous oxide is leached into water, it is oxidized; thus, the predominant form of copper is the active substance, cupric ion, Cu^2+^. Copper is known to be an essential element for photosynthesis and respiration [18,19]. It is required as a cofactor for a number of enzymes that are involved in various cellular processes [20]. However, excessive Cu has negative effects on the growth and productivity of photosynthetic organisms [21,22]. It results in damage of lipids, proteins, DNA, and other cytoplasmic molecules [23,24].

Inorganic zinc is often used in combination with copper to increase the overall toxicity of the formulation or to facilitate the leaching process. It is considered to increase the release rate of the copper included in the paint [13]. Zinc is also used to improve coating performance and prevent erosion. Organic Zn-based booster biocides are also added to the paints to enhance the antifouling effectiveness. One of them is zineb (zinc ethylenebis-(dithiocarbamate)), which has been found to be the growth inhibitor for fouling species, including freshwater and marine autotrophs. It is proposed for use against red and green algae, diatoms, and invertebrates [25]. Zineb acts as a general inhibitor of metabolic pathways through interactions with thiol (-SH) groups within metabolically active proteins [25]. Zineb decomposes when exposed to moisture, and the products of its decomposition are ethylenethiuram monosulfide, zinc sulfide, carbon disulfide, and ethylenethiourea [26]. The toxicity of the biocides, including Cu_2_O and zineb, the mechanism of action, persistence, fate, and behaviour are not fully understood. Few data have been published on the toxicity of copper and zineb-based AF paints against phototrophic organisms. Taking into account the relevant lack of knowledge, assessing the overall effect of these biocides is challenging and crucial.

Cyanobacteria are the only group of prokaryotes capable of photosynthesis and respiration simultaneously in the same compartment, and many cyanobacterial species are able to fix nitrogen. Therefore, they can survive and prosper under a wide range of environmental conditions. Hardiness of cyanobacteria makes them spread out in almost all ecological niches, including fresh and salt water or harsh environmental areas [27]. Cyanobacteria are currently used as an effective species for the mitigation of a diverse range of environmental contaminants, including metals [28]. Different heavy metals affect cyanobacterial cells differently, and the response triggered to cope with these metals is also quite distinctive [20,22]. Cyanobacteria are among the organisms that are particularly sensitive to copper [29]. A unicellular, nitrogen-fixing cyanobacterium *Cyanothece* shows a highly selective affinity for Cu(II) and is considered a very promising biosorbent for the selective removal of Cu(II) from aqueous solutions. *Cyanothece* also reveals a very rapid uptake of Cu(II), and the saturation of the metal sorbing capacity does not exceed 30 min [30]. Therefore, *Cyanothece* represents an excellent experimental model organism for the study of the metabolic events under heavy metal exposure. Exposure of *Cyanothece* cells to Cu^2+^ results in the adjustment of the metabolic rate, including O_2_ evolution, CO_2_ fixation, and N_2_ assimilation [22], but a physiological response is mostly dependent on the time exposure and/or copper ion concentration.

Among the bioculture monitoring techniques, the non-invasive approaches are of great importance. Non-invasive optical techniques, including chlorophyll (Chl) fluorescence and infrared thermography, provide essential tools to monitor the early response of photosynthetic organisms to environmental stresses [31,32]. Phototrophic samples have the advantage of possessing chlorophyll, a green pigment-protein molecule, which is essential for photosynthesis. Light energy absorbed by chlorophyll molecules can undergo one of three fates: (i) it can be used to drive photosynthesis, (ii) excess energy can be dissipated as heat, or (iii) it can be re-emitted as chlorophyll fluorescence. Although the total amount of Chl fluorescence is very small (up to 5% of total light absorbed), this non-invasive technique provides a powerful tool for photosynthetic performance in plants, algae, and cyanobacteria. Analysis of Chl fluorescence can be used to obtain quantitative estimates of the quantum yield of photochemistry in photosystem II (PSII) [33]. One of the primary applications is determining the photosynthetic activity of photosystem II in vivo [34,35,36]. Illumination of dark-adapted samples leads to an increase in fluorescence from a minimum (F_0_) to a maximum (F_M_) level, which decreases thereafter and depends on the physiological status of the sample and the experimental conditions. The initial increase in fluorescence is usually the result of the reduction of electron carriers in the photosynthetic membranes and is correlated with the redox state of the Q_A_, the primary quinone acceptor of the photosynthetic reaction centre. When Q_A_ is oxidized, the reaction centre is able to utilize the light energy harvested by chlorophylls for charge separation, and the fraction of excitation lost to fluorescence is minor, so that the fluorescence yield remains low. As the quinone pool gradually reduces, the reaction centre is unable to undergo stable charge separation. The fraction of excitation lost to fluorescence is high, and the fluorescence yield gradually rises to the maximum fluorescence yield [33,37].

In this research, *Cyanothece* cultures were subjected to antifouling coatings containing 21.4% Cu_2_O and zineb (in varying concentrations) as the main booster biocides. We used an in vivo chlorophyll *a* fluorescence technique to measure the dynamics of the physiological response of *Cyanothece* to short-term paint exposure (32 h). Non-invasive fluorescence analysis, along with the screening of cell adhesion to AF coatings, may be useful for assessing the dynamics of the release of toxic agents into aquatic media at the initial phase of fouling. Environmental protection is very important in times of significant industrialisation. This study presents a novel approach to the analysis of the toxicity of antifouling paints. This is important in the context of developing new surface protective coatings that show potential to reduce the release of highly toxic substances, such as copper, zinc, etc., into the environment. Our proposed approach can contribute to optimising the amount of toxic substances released so as not to reduce the effectiveness of surface protection while protecting the aquatic ecosystem.

## 2. Results

### 2.1. Bioaccumulation of Elements in Cyanobacterial Cells

In this study, AF coatings containing copper dioxide and zineb as the main biocidal agents were investigated. The AF capacity was tested against cyanobacteria by focusing on the inhibition of the photosynthetic activity. Eight different materials were used (i) containing Cu_2_O (21.4%) (J1–J4) and zineb (at varying concentrations, Table 1), and (ii) copper-free coatings (J5–J8) formulated with zineb biocide (J5–J7; see Table 1). Each coating had the same size and roughness and was immersed in cyanobacterial culture for 32 h.

We carried out the ICP-OES analysis and focused on determining the concentration of Cu^2+^ and Zn^2+^ as the main constituent elements of biocides, i.e., copper dioxide and zineb incorporated into AF paints. The average copper concentration for *Cyanothece* cultures exposed to J1–J4 coatings was 5.336 ± 0.640 g/kg DW, and the zinc concentration ranged from 101.60 ± 12.192 mg/kg DW (J3, J7) to 329.40 ± 39.528 mg/kg DW (J1, J5). Bioaccumulation of zinc for *Cyanothece* suspensions treated with J2 and J6 coatings was also confirmed, where the Zn^2+^ content was 216.20 ± 21.155 mg/kg DW. The ICP-OES analysis also allowed the determination of other elements. Interestingly, the most pronounced changes in element content were found in cyanobacterial cells exposed to copper-based paints. In these cultures, compared to control (i.e., the culture that was not exposed to AF paints), we observed an increase in the levels of iron (1.33-fold) and manganese (2.32-fold). Interestingly, those elements, such as copper and zinc, are cofactors of antioxidant enzymes, such as iron superoxide dismutase (FeSOD) and manganese superoxide dismutase (MnSOD). We also observed an increase in the content of phosphorous (1.60-fold), barium (9.90-fold, copper-based coatings; 28-fold, copper-free coatings), vanadium (1.2-fold), calcium (1.24-fold) and aluminum (2.20-fold).

### 2.2. Response of the Photosynthetic Activity to Antifouling Coatings in Cyanothece sp. ATCC 51142

**Figure 1:**
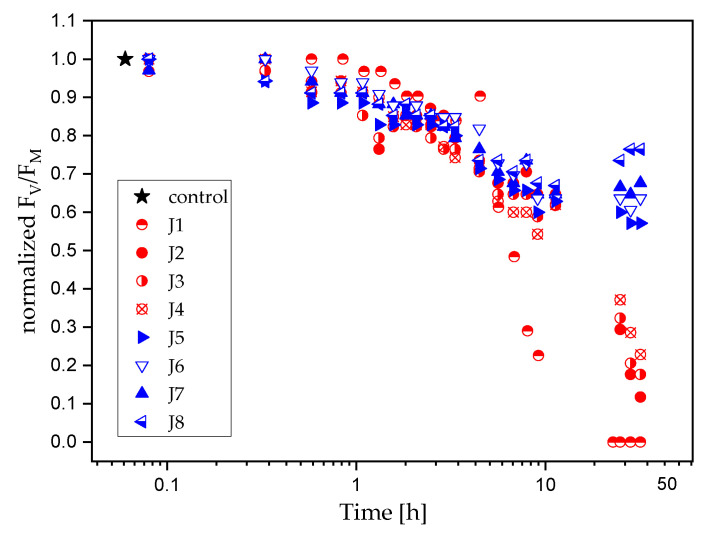
Semi-logarithmic graph, which presents changes in the maximum quantum yield of PSII photochemistry (F_V_/F_M_) of *Cyanothece* cells: (i) not exposed to antifouling paints (control culture, star symbol), (ii) exposed to copper-based (circle symbols), and (iii) copper-free (triangular symbols) antifouling coatings. For each treatment, the F_V_/F_M_ ratio was normalized to the range [0, 1], where 0 and 1 correspond to the minimum and maximum values of F_V_/F_M_, respectively.

To reveal changes in the activity of photosystem II under Cu_2_O and/or zineb action, the maximum quantum yield of PSII photochemistry (Φ_PSII_) was measured and expressed as a relation of F_V_/F_M_ (Figure 1).

The results show that all AF coatings had an important inhibitory effect on the photosynthetic activity of *Cyanothece* cells. However, the magnitude of photosynthetic inhibition varied between paints, reaching its highest value for the J1 coating. The activity of photosystem II decreased significantly in all cultures exposed to AF coatings; total inhibition could be observed for the J1 paint. This paint contained a dicopper oxide (21.4%) and the highest amount of zineb (12.8%). Almost 80–90% of the diminishing of F_V_/F_M_ was observable for treatment with J2, J3, and J4 paints. Photosynthetic activity was impacted to a lower extent for non-copper-based coatings, and was reduced by 43% (J5), 36% (J6), and 32% (J7), as compared to the control culture. Furthermore, after 24 h of exposure, the recovery of photosynthetic activity was observed, where the F_V_/F_M_ recovered was 23% less than the controls in the case of J8 coating. J8 was the only paint tested that did not contain active biocide agents, i.e., zineb and Cu_2_O. Under the exposure of J8, Φ_PSII_ reached 65% of its total (initial) value.

**Figure 2:**
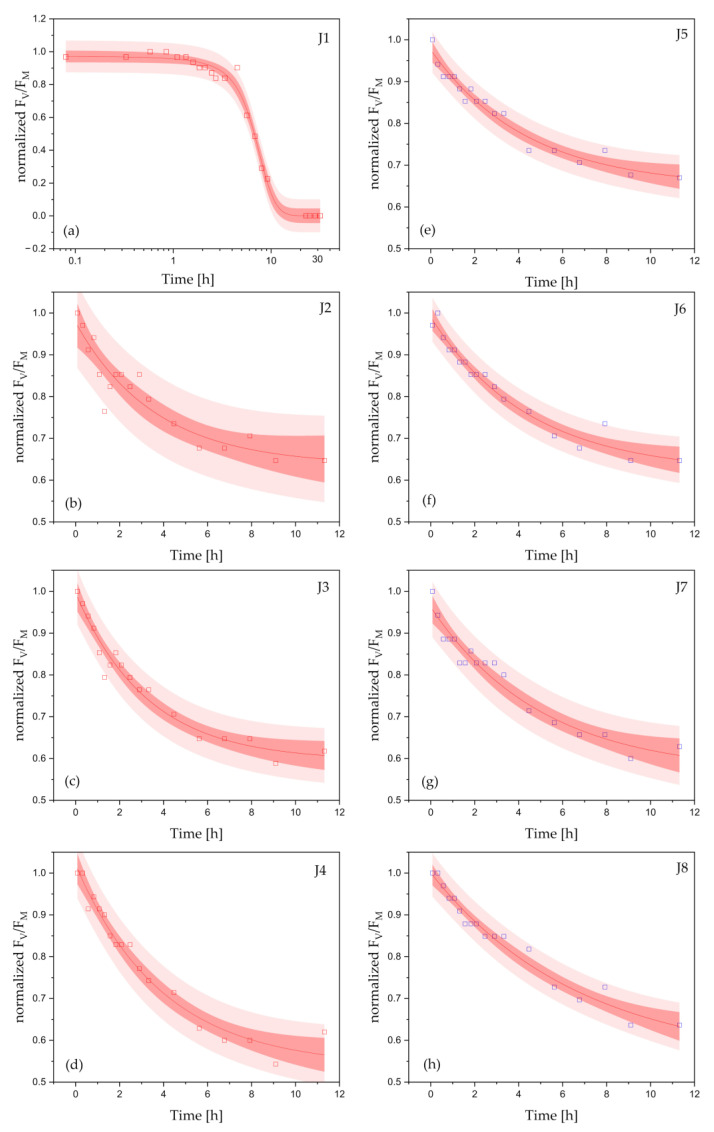
Fluorescence courses (F_V_/F_M_) fitted using a sigmoid (**a**) or mono-exponential (**b**–**h**) function, approximate the initial responses of *Cyanothece* to copper-based antifouling paints 12 h after exposure to J1 (**a**), J2 (**b**), J3 (**c**), J4 (**d**), J5 (**e**), J6 (**f**), J7 (**g**), and J8 (**h**). Each graph (**a**–**h**) shows the fitted fluorescence data along with the 95% prediction (light red surfaces) and confidence (dark red surfaces) bands. For each treatment, the F_V_/F_M_ ratio was normalized to the range [0, 1], where 0 and 1 correspond to the minimum and maximum values of F_V_/F_M_, respectively. For better readability of the fitted curves, the range of values on the ordinate axis in (**b**–**h**) is different than in (**a**).

**Figure 3:**
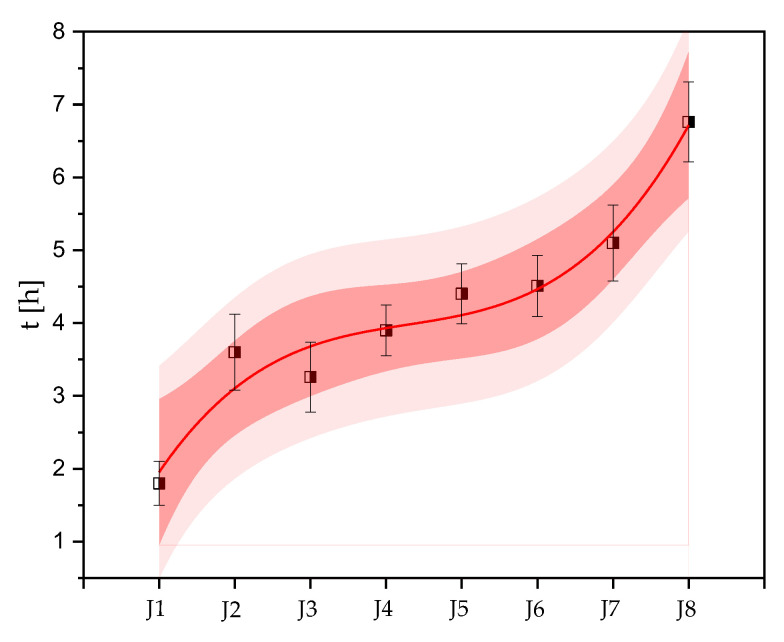
Variability of time constants estimated for *Cyanothece* exposed to antifouling coatings. Data are shown along with 95% prediction (light-red surfaces) and confidence (dark-red surfaces) bands.

In this research, the examined coatings were immersed in cyanobacterial cultures for 32 h. To further probe the dynamics of the AF action against cyanobacteria, we evaluated the kinetics of the F_V_/F_M_ changes in *Cyanothece* exposed to AF coatings. The data showed that the gradual decrease in the maximum PSII photochemistry (F_V_/F_M_) was mainly caused by biocides (Cu_2_O and Zineb) released from the AF paints. The kinetics of the response of *Cyanothece* to AF paints are presented in Figure 2 (copper-based coatings, a–d; copper-free coatings, e–h). Each kinetics evaluated for the coating J1 (Figure 2a), J2 (Figure 2b), J3 (Figure 2c), and J4 (Figure 2d) revealed a sigmoid (Figure 2a) or exponential (Figure 2b–d) decay and showed the significant differences in the courses between cyanobacterial cultures exposed to the investigated paints. In turn, all the courses assessed for *Cyanothece* exposed to copper-free coatings (J5–J8, Figure 2e–h) showed an exponential decline. The experimental data, which show changes in F_V_/F_M_ over time, were fitted using a function (i) (Figure 2a):

*y_J_*_1_(*t*) = [1 + *exp*((*t* − *t*_0_)/*t_J_*_1_)]^−1^, (1)

or (ii) mono-exponential function (Figure 2b–h):

*y_J_*(*t*) = *y*_0_ + *y_m_* [*exp*(−*t*/*t_J_*)], (2)

where *t_J_*_1_ and *t_J_* correspond to the time constant, *t*_0_ is the time for which the function *y_J_*_1_(*t*) reaches a half of its value, *y*_0_ is an amplitude at the end of the *y_J_*(*t*) decay, and *y_m_* stands for a maximum amplitude. The bands (Figure 2a–h) show 95% confidence (dark red) and prediction (light red) intervals. Analysis of fluorescence parameters revealed that within copper-based paints, the time constants increased with decreasing zineb content, reaching the lowest value (1.73 ± 0.30 h) for J1, which contained the highest amount of Cu_2_O and zineb biocides (Figure 3). J1 showed the highest toxicity against *Cyanothece* among all the coatings studied. After *t*_0_ = 6.75 ± 0.22 h, *Cyanothece* cells revealed a half-reduction in photosynthetic activity. For the other coatings tested (J2–J4), the time constants were: 3.60 ± 0.52 h (J2), 3.26 ± 0.48 h (J3), and 3.90 ± 0.35 h (J4). Interestingly, *Cyanothece* exposed to copper-free coatings revealed the significant deceleration in F_V_/F_M_ courses. This resulted in an increase in time constants compared to the J1–J4 paints. The time constants determined for cultures under exposure of copper-free paints were: 4.40 ± 0.41 h (J5), 4.51 ± 0.42 h (J6), 5.10 ± 0.52 h (J7). The highest value (*t_J_* = 6.75 ± 0.55 h) was assessed for J8 coatings. Within all coatings studied, J8 was the only paint not formulated with Cu_2_O and zineb.

**Figure 4:**
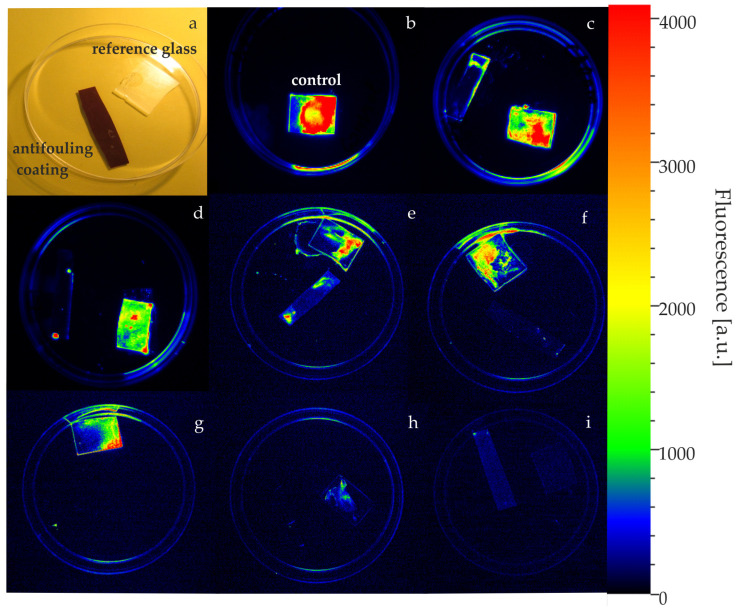
The biofilm of cyanobacterial cells visualized using a fluorescence imaging system in the initial stage of overgrowth on the surfaces of antifouling paints and glass pieces, placed as a reference (**a**). The control culture adhered to the glass is shown in (**b**). The red colour corresponds to the intensity of fluorescence, and thus to the number of cells. The images (**c**–**e**) show the layer of *Cyanothece* biofilm on the copper-free coatings. Very weak growth (**f**,**g**) or no growth (**h**,**i**) of cyanobacteria was observed on copper-based antifouling paints.

The efficiency of AF paints against monoculture cyanobacteria biofilm was also visualized using a fluorescence imaging system. The initial phase of overgrowth on the surfaces of antifouling paints is shown in Figure 4. The red colour corresponds to the intensity of fluorescence, and thus the viable cell counts. For adhered cells, small pieces of different coatings were placed in Petri dishes with glass used as a reference (Figure 4a). A control culture of *Cyanothece* adhered to the glass surface is presented in (Figure 4b). The images (Figure 4c–e) show the *Cyanothece* biofilm layer on the copper-free coatings.

Very weak growth (Figure 4f,g) or no growth (Figure 4h,i) of cyanobacteria was observed on copper-based antifouling paints. At this initial stage of fouling, the cyanobacterial biofilm was observable, only when using the camera, as a fluorescent image.

## 3. Discussion

In this research, we used in vivo chlorophyll *a* fluorescence to test the photosynthetic activity of *Cyanothece* cultures exposed to copper-based and copper-free AF coatings formulated with zineb. The efficiency of these coatings was assessed by (i) the release of active biocidal ions (mainly, Cu^2+^, Zn^2+^) from the surfaces of the coatings into the aqueous medium and (ii) the contact of cyanobacterial cells with the coated surfaces.

This study has shown that the AF coatings tested (i) inhibited (J1–J4) or (ii) notably limited (J5–J8) the photosynthetic activity of *Cyanothece* cells. The copper present in the J1–J4 paints in the form of copper dioxide (Cu_2_O) was the main biocide. The study showed that the antifouling efficiency of copper was the most pronounced. This is in agreement with [16] that copper is an effective biocide that can affect aquatic organisms and cause environmental concerns. Copper is a trace element that is required at miniscule levels for the proper functioning of all organisms. However, it can be toxic at higher concentrations, with a lethal concentration value ranging from 5–10^5^ µg/L. In this study, the copper concentration determined in cyanobacterial cultures was 9.01 ± 1.05 mg/L. Cyanobacteria are one of the most sensitive species to copper toxicity [38,39]. However, the lethal threshold may differ between species since organisms have different mechanisms to cope with and process copper [16]. Furthermore, the previous study presented by [22] has shown that Cu^2+^ exposure causes a decrease in the metabolic rate in *Cyanothece* cells, CO_2_ fixation, and N_2_ assimilation, and also leads to increased reactive oxygen species. Under Cu^2+^ exposure, the disintegration and disorganization of the thylakoid membrane was observed. Acute exposure for 24 h to Cu^2+^ was the condition that promoted a greater impairment in O_2_ evolution in *Cyanothece*. Other studies have shown that under Cu^2+^ exposure, the evolution rate of O_2_ ceases in other cyanobacteria [40,41]. Interestingly, in study [22], the concentration of copper did not exceed 1 mg/L, and was 9-fold lower than that used in J1–J4 paints.

This research shows a mechanistic action of the AF compounds (Cu_2_O and zineb). Zineb, an organic booster biocide, is currently widely used for AF applications. In our study, zineb contributed to an enhanced effectiveness of copper-based and copper-free AF coatings. This is in agreement with [42,43] that the enhanced toxicity of AF paints could be attributed to zinc leached from AF paints. In particular, it is noticeable when comparing AF paints J1 (21.4% Cu_2_O, 12.8% zineb) and J4 (21.4% Cu_2_O, 0% zineb). Exposure of cyanobacterial cells to J1 caused irreversible damage (F_V_/F_M_ = 0) to photosynthetic activity, while the action of J4 resulted in an 80% reduction in the initial F_V_/F_M_. A similar effect was observed in the case of copper-free AF coatings. Exposure of *Cyanothece* to J5 coating (containing 12.8% zineb) revealed a 40% decrease in initial photosynthetic activity and was almost 20% lower than that measured for the cells treated with J8 coatings. An important finding is the recovery of photosynthetic activity observed for *Cyanothece* under exposure to the J8 coating, which confirmed the reversibility of the photosynthetic inhibition effect. The J8 coating did not act as a biocide and the recovery of F_V_/F_M_ was revealed 24 h after exposure. Within all coatings studied, formulated with copper (in the form of Cu_2_O) as an active biocide, we observed an irreversible inhibition of photosynthetic capacity. Therefore, this research confirms that Cu_2_O is the most effective biocide against *Cyanothece*, regardless of the amount of zineb incorporated into the tested paints. Exposure of cyanobacterial cells to a J1 coating formulated with the highest concentration of Cu_2_O and zineb, resulted in an inhibition of photosynthetic activity. This irreversible inhibition could be due to damage to photosystem II as a consequence of the generation of reactive oxygen species [22,44].

To study the dynamics of an AF effectiveness of coatings, we proposed a novel approach in fluorescence analysis based on the determination of time constants (*t_J_*) of changes in F_V_/F_M_. Previous studies [45,46,47] have demonstrated the use of chlorophyll *a* fluorescence in the evaluation of photosynthetic activity of photosynthetic aquaculture, but as far as we are concerned, ours is the first study that sheds new light on the evaluation of efficacy of AF paints. This approach focused on the assessment of the dynamics of changes in photosynthetic activity by means of determining the characteristic time constants. Time constants were estimated by fitting the F_V_/F_M_ dependencies over time using an exponential and/or sigmoid function. This analysis showed that under the action of copper-based and copper-free AF coatings formulated with zineb, the changes in time constants evaluated for *Cyanothece* can be approximated by the polynomial function. The calculated time constants correspond to the dynamics of toxicity of the studied paints and reveal the lowest values for the copper-based coatings. Interestingly, the *t_J_* values increase as the AF efficiency of coatings decreases. The presence of zineb in copper-based coatings (J1–J3) has strongly increased the dynamics of action in AF paints. As mentioned above, a reversible inhibition of photosynthesis was observed for the tested copper- and zineb-free (J8) coating. In this case, the calculated time constant of changes in F_V_/F_M_ showed the highest value among all the paints tested, which was almost four times higher than the *t_J_* obtained for the J1 coating.

The settlement of micro- and macro-organisms is a natural phenomenon that occurs continuously and vigorously on immersed surfaces. There is a wide range of organisms involved in this process at different trophic levels, including bacteria, phototrophic micro- and macro-organisms, and protozoa [48]. The fouling process is complex and follows the specific phases characteristic of the fouler, e.g., molecular fouling, microfouling, particulate fouling, and macrofouling [49]. The settlement of unicellular photosynthetic organisms (oxygenic photoautotrophs) belongs to one of the fastest stages of colonization that occur during the first 24 h after immersion [50]. In this research we visualised the *Cyanothece* cells adhered to AF coatings in the initial stage of biofouling using a fluorescence imaging system. We monitored cyanobacterial overgrowth 24 h after exposure to AF paints. The layer of *Cyanothece* biofilm was detected only on the copper-free coatings. No visible settlement was found under exposure to copper-based AF paints.

## 4. Materials and Methods

### 4.1. Cyanobacterial Culture Exposed to Antifouling Coatings

*Cyanothece* sp. *strain 51142* purchased from the American Type Culture Collection (ATCC), was grown in artificial seawater medium-ASP2 in the presence of nitrate in a controlled environment chamber AlgaeTron AG 130 (Photon Systems Instruments, Drásov, Czech Republic) under a light intensity of 50 µE m^−2^·s^−1^ and a temperature of 25 °C. Cells were cultivated in sterile Erlenmeyer flasks with shaking at 125 rpm for 7–10 days. After the culture reached an optical density of approximately 0.3, small (3 × 1.5 × 0.4 cm) pieces of antifouling coatings were immersed in the cyanobacterial suspension and incubated for 32 h prior to fluorescence measurements. Antifouling coatings for testing were provided courtesy of PPG Industries, Inc., Pittsburgh, PA, USA. We studied non-copper coatings and coatings that contained copper (21.4%) and zineb (in varying concentrations from 0 to 12.8%) as active biocide agents (Table 1).

### 4.2. Element Analysis

Before analysis, cell cultures were filtrated using MCE membrane filters with a pore diameter of 0.45 µm. The solutions after filtration were acidified by adding concentrated HNO_3_ Suprapur (Sigma-Aldrich, Saint Louis, MO, USA) to a pH of approximately 1–2. The total concentration of elements was determined using the ICP-OES technique according to the 11,885 ISO standard. The iCAP PRO XP spectrometer from Thermo Fischer Scientific (Waltham, MA, USA) was used. Analytical lines 224.700 nm, 213.856 nm, 308.215 nm, 455.403 nm, 315.887 nm, 259.940 nm, 257.610 nm, 177.495 nm, and 292.402 nm were selected for the determination of Cu, Zn, Al, Ba, Ca, Fe, Mn, P, and V, respectively. The quantification limits for all elements ranged from 0.005 µg/L to 0.01 µg/L. The details can be found in [51,52]. The residuals remaining on the filters were digested in a microwave mineraliser UltraWAVE (Milestone Srl, Sorisole BG, Italy) in the presence of concentrated HNO_3_. The digestion was carried out at a temperature of 230 °C and a maximum pressure of 80 bar for 25 min. The digests were diluted with deionised water and analysed in the same way as the solutions after filtration.

### 4.3. Measure of Photosynthesis

In this study, the impact of coatings on photosynthetic activity was estimated using in vivo chlorophyll *a* fluorescence measured on cell suspension. The maximum quantum yield of photosystem II photochemistry (Φ_PSII_) was measured using an AquaPen AP 100 fluorometer (Photon Systems Instruments, Drásov, Czech Republic). Two milliliters of cell suspension adjusted to an optical density of 0.3 was dark-adapted for 15 min, and then chlorophyll fluorescence was recorded. Fluorescence measurements were monitored at 15 min intervals for 32 h and experiments were carried out to: (i) evaluate the AF activity of different coatings against *Cyanothece* due to the release of the main two biocides (dicopper oxide and zineb); (ii) measure the photosynthetic activity (and its recovery) of cyanobacterial cells affected by AF treatment. Saturating and measuring light intensity was 3000 and 0.05 µmol (photons) m^−2^·s^−1^, respectively. Light was provided by blue (455 nm) and red (630 nm) light-emitting diodes (LEDs). Φ_PSII_ was determined as F_V_/F_M_, where F_V_ = (F_M_ − F_0_) is variable fluorescence, and F_M_ stands for the maximal fluorescence recorded in the dark-adapted state [33]. All experiments were performed at room temperature with at least three repetitions in complete darkness.

### 4.4. Imaging of a Biofilm on Antifouling Coatings

The fluorescence-based screening technique was used to detect cyanobacterial biofilm at the initial fouling phase. The biofilm was monitored using the FluorCam FC 800-MF pulse-amplitude modulated chlorophyll fluorometer (PAM) (Photon Systems Instruments, Drásov, Czech Republic). Excitation light was produced by blue (455 nm) LEDs. Actinic light intensity was 2000 µmol (photons) m^−2^·s^−1^. A plate with the algal biofilm was put onto an imaging chamber to perform a chlorophyll fluorescence measurement. Fluorescence data were elaborated by FluorCam 7 software. Before measurements, cyanobacterial cultures were dark-adapted for 15 min.

### 4.5. Statistical Analysis

The statistics were analysed and the data evaluated using Origin Professional software version 2019b (Origin-Lab; Northampton, MA, USA). Statistically significant differences between the fluorescence courses of cyanobacterial cultures exposed to AF coatings were determined using the Mann-Whitney *U* test.

## 5. Conclusions

In conclusion, copper- and non-copper based antifouling coatings formulated with zineb were tested to study the potential inhibition of photosynthesis in cyanobacterial culture. Photosynthetic measurements were performed using in vivo chlorophyll *a* fluorescence. The inhibition effect was observed when (i) cyanobacteria were exposed to Cu^2+^ and Zn^2+^ released from AF paints into the aqueous medium, or (ii) cells were in contact with the coated surfaces. The presence of zineb in copper-based AF coatings improved the inhibition effect. The study shows that copper-zineb-based coatings can reveal a higher harmful impact at the ecosystem level. Therefore, the analysis we proposed along with the screening fluorescent results may be useful in evaluating the initial antifouling dynamics against phototrophic aquacultures. Non-invasive fluorescence analysis and estimation of the dynamic changes in the physiological response during early overgrowth are key to ecosystem conservation, which should be aimed at developing more environmentally friendly AF coatings.

## Figures and Tables

**Table 1:** Composition in wt% of antifouling paints containing Cu_2_O and zineb booster biocides.

Paint Label	Cu_2_O [%]	Zineb [%]
J1	21.4	12.8
J2	21.4	8.6
J3	21.4	4.3
J4	21.4	0
J5	0	12.8
J6	0	8.6
J7	0	4.3
J8	0	0

## Data Availability

The data presented in this study are available in article.

## Abbreviations

AF, antifouling; Chl, chlorophyll; F_0_ (F_M_), the minimum (maximum) chlorophyll *a* fluorescence in the dark-adapted state; LED, light emitting diode; PSII, photosystem II; Q_A_, primary quinone acceptor of photosystem II.
